# Hair cortisol as a hypothalamic-pituitary-adrenal axis biomarker in pregnant women with asthma: a retrospective observational study

**DOI:** 10.1186/s12884-016-0962-4

**Published:** 2016-07-20

**Authors:** Laura Smy, Kaitlyn Shaw, Ursula Amstutz, Anne Smith, Howard Berger, Bruce Carleton, Gideon Koren

**Affiliations:** Division of Clinical Pharmacology and Toxicology, The Hospital for Sick Children, Toronto, ON Canada; Pharmaceutical Sciences, Leslie Dan Faculty of Pharmacy, University of Toronto, Toronto, ON Canada; Child & Family Research Institute, Vancouver, BC Canada; Division of Translational Therapeutics, Department of Pediatrics, University of British Columbia, Vancouver, BC Canada; University Institute of Clinical Chemistry, Inselspital Bern University Hospital, University of Bern, Bern, Switzerland; Department of Obstetrics & Gynecology, St. Michael’s Hospital, Toronto, ON Canada; Pharmaceutical Outcomes Programme, Child & Family Research Institute, 950 W 28th Avenue, Vancouver, BC V5Z 4H4 Canada

**Keywords:** Hair cortisol, Pregnancy, Asthma, Biomarker, HPA axis, Adrenal suppression

## Abstract

**Background:**

Cortisol is a hormone involved in many physiological functions including fetal maturation and epigenetic programming during pregnancy. This study aimed to use hair cortisol as a biomarker of chronic inhaled corticosteroid (ICS) exposure and assess the potential effects of asthma on the hypothalamic-pituitary-adrenal (HPA) axis in pregnant women. We hypothesized that pregnant women with asthma treated with ICS would exhibit lower hair cortisol concentrations, indicative of adrenal suppression, compared to women with asthma not using ICS and women who do not have asthma.

**Methods:**

We performed an observational retrospective cohort study. Hair samples were analyzed from pregnant women with asthma, with (*n* = 56) and without (*n* = 31) ICS treatment, and pregnant women without asthma (*n* = 31). Hair samples were segmented based on the growth rate of 1 cm/month and analyzed by enzyme immunoassay to provide cortisol concentrations corresponding to preconception, trimesters 1–3, and postpartum. Hair cortisol concentrations were compared within and among the groups using non-parametric statistical tests.

**Results:**

Hair cortisol concentrations increased across trimesters for all three groups, but this increase was dampened in women with asthma (*P* = 0.03 for Controls vs. ICS Treated and Controls vs. No ICS). ICS Treated women taking more than five doses per week had hair cortisol concentrations 47 % lower in third trimester than Controls. Linear regression of the third trimester hair cortisol results identified asthma as a significant factor when comparing consistent ICS use or asthma as the predictor (F_(1, 25)_ = 9.7, *P* = 0.005, R^2^_adj_ = 0.257).

**Conclusions:**

Hair cortisol successfully showed the expected change in cortisol over the course of pregnancy and may be a useful biomarker of HPA axis function in pregnant women with asthma. The potential impact of decreased maternal cortisol in women with asthma on perinatal outcomes remains to be determined.

**Electronic supplementary material:**

The online version of this article (doi:10.1186/s12884-016-0962-4) contains supplementary material, which is available to authorized users.

## Background

Cortisol has many important functions in the body including metabolism, regulation of blood pressure, and roles in the inflammatory and stress responses. When required, the hypothalamus and pituitary gland are activated to release a series of hormones that signal the adrenal cortex to release cortisol. Asthma is a common chronic inflammatory disease for which the recommended therapy for long-term control is inhaled corticosteroids (ICS) [1]. ICS bind to glucocorticoid receptors in lung epithelial cells and reduce airway inflammation. Binding also mimics endogenous cortisol and consequently initiates a negative feedback resulting in decreased cortisol release from the adrenal glands. While generally believed to confer less systemic exposure than systemic corticosteroids, in severe cases, the use of ICS may cause adrenal insufficiency or crisis [2, 3].

Classically, the matrices used to measure a patient’s cortisol level are blood, saliva, or urine. However, these matrices do not account for the diurnal nature of cortisol secretion and only reflect the point in time when sampling is conducted [4]. More recently, hair analysis has emerged as a viable alternative for cortisol detection. Hair cortisol levels correlate with a 24-h urine sample (*r* = 0.33, *p* = 0.04) and multiple saliva samples collected over 7 days (*r* = 0.41, *p* = 0.03) [4, 5]. Since hair grows on average one centimeter per month (cm/mo) ([6], p. 2), the cortisol detected in a one-centimeter hair segment represents the average cortisol level over the corresponding one-month period.

Hair cortisol has previously been used to assess the effects of various medical conditions on the hypothalamic-pituitary-adrenal (HPA) axis, including psychological and physical stressors. It is already known that cortisol concentrations increase in relation to a stressful event, and these changes were reflected in the hair of children fearful of beginning school and individuals who recently experienced a traumatic event [7–9]. Similarly, hair cortisol concentrations in patients diagnosed with Cushing syndrome corresponded to the characteristic increased endogenous cortisol concentrations, with 86 % sensitivity and 98 % specificity for the detection of cyclic Cushing’s syndrome [10, 11]. Recently, we examined hair cortisol of children with asthma due to concerns of potential adverse effects from long-term ICS therapy [12]. Our results showed a 55 % decrease in hair cortisol concentration during ICS therapy compared to prior to ICS therapy suggesting significant ICS-induced HPA axis suppression and providing further support for using hair as a matrix for measuring cortisol.

In 2001 in the United States, the prevalence of asthma in pregnancy was 8 % [13]. Only one study has examined hormone concentrations, including cortisol, among pregnant women with asthma with and without ICS treatment as compared to pregnant women without asthma and found no difference among the three groups [14]. Given that cortisol is a vital factor in fetal lung, gastrointestinal tract, kidney, and thyroid maturation [15], our objective was to use hair cortisol as a biomarker to investigate cortisol changes over the course of pregnancy in the context of potential adverse effects of ICS on the HPA axis in pregnant women with asthma. We hypothesized that pregnant women with asthma treated with ICS would exhibit lower hair cortisol concentrations, suggestive of adrenal suppression, compared to women with asthma not using ICS and women who do not have asthma.

## Methods

### Study design, participants, and ethics

We performed a retrospective observational cohort study. Three groups of pregnant women were recruited from June 2012 to December 2014: women with ICS-treated asthma (ICS Treated), and two comparison groups consisting of women without asthma (Controls) and a disease-matched group of women with asthma not treated with ICS (No ICS). The sample size was not formally determined due to the unavailability of data on hair cortisol concentrations in pregnant women with and without asthma when the protocol was created. Participants were recruited in person in obstetric clinics at the British Columbia Women’s Hospital and Health Centre in Vancouver, British Columbia, and St. Michael’s Hospital in Toronto, Ontario. Additionally, women were recruited through the Hospital for Sick Children’s Motherisk Program teratology information service via telephone and mail. All pregnant women were eligible provided they could read and understand English, did not use any corticosteroid products on their scalp, or have any known medical conditions characterized by high cortisol levels such as Cushing’s syndrome. Women were recruited at any time during pregnancy up to 6 months postpartum. Research ethics board approval was obtained from each institution and informed written consent was obtained from all participants.

### Data collection

Relevant clinical information was obtained through medical record review and/or patient interview and included asthma and ICS treatment history and concomitant medications. Factors reported to affect hair cortisol levels, such as frequency of hair washing, days since last washing and chemical treatment (color or relaxer), were also collected [16]. Additionally, the Perceived Stress Scale (PSS), a validated tool to assess stress levels experienced in the previous month, was administered to women enrolled in Ontario [17].

### Hair sample collection and analysis

A lock of hair approximately 3 mm thick (equivalent to one-half the diameter of a pencil) was cut from the vertex posterior region of the head as close to the scalp as possible. Based on an average growth rate of 1 cm/mo, each hair sample was further cut into segments 2–3.6 cm in length (the majority were 3 cm long) to correspond to preconception (PC), trimesters 1–3 (T1, T2, T3), and immediate postpartum (PP) time points as available depending on the hair length and collection date ([6], p. 2). Day 0 of each hair sample was considered fourteen days prior to date of hair collection to account for the fact that 10–14 days of hair resides below the scalp ([6], p. 35). Segmented hair samples were processed for hair cortisol extraction as previously reported [18], with a few minor modifications. In brief, each sample of 10–25 mg of hair was washed twice with isopropanol and allowed to dry, and then finely minced with scissors and extracted overnight in methanol. After removing all of the supernatant, samples were dried under N_2_ at 37 °C, reconstituted with 125 μL of phosphate buffered saline, and vortexed for one minute. Initially, samples were reconstituted with 250 μL, but this resulted in some samples having results below the quantitation limit (0.33 pmol/mL or 0.12 ng/mL).

The samples were analyzed using the Salimetrics High Sensitivity Salivary Cortisol Enzyme Immunoassay (EIA) kit (Salimetrics, Philadelphia, PA) following the manufacturer’s instructions. All of the % cross-reactivity reported by the manufacturer is less than 0.6 % for other steroid hormones, such as prednisolone, prednisone, or cortisone, except for dexamethasone, which was 19.2 %. Additionally, the cross-reactivity was determined for each of the ICS available in Canada using an 8000 ng/mL solution run six times on two different EIA plates. The results were not detectable for fluticasone propionate, budesonide and ciclesonide, 0.01 % for beclomethasone dipropionate, and 0.03 % for mometasone furoate. Hair cortisol concentrations measured using this kit have been correlated to two different liquid chromatography-mass spectrometry methods with a Spearman rho of ≥ 0.92 (*P* < 0.0001) [19].

In addition to the quality control material included with the kit, a pooled in-house hair sample was run as a third quality control and the results were evaluated using Westgard Rules for acceptance or rejection. The results of the EIA were considered acceptable if two of three control values were within expected range. Based on the coefficients of variation for the participant samples, the average intra-day coefficient of variation was 6.3 %. The average inter-day coefficients of variation were 1.5 and 4.5 % for the high and low quality control, respectively. Because all samples are run in duplicate with EIA analysis, any duplicates that had a coefficient of variation greater than 15 or 20 % for results greater than or lower than approximately 3 pmol/mL (1 ng/mL), respectively, were reanalyzed or reprocessed based on sample availability. Cortisol concentrations are reported as a ratio to the hair sample weight (pmol/g).

### Statistical analysis

Statistical analysis was performed using Graphpad Prism software, version 5.0c (Graphpad Software, Inc., La Jolla, CA) and SPSS, version 22.0 (IBM Corporation, New York, NY). Comparisons between the groups for hair cortisol concentrations, demographic information, and clinical information were performed using one-way analysis of variance with Tukey’s multiple comparison test, the Kruskal-Wallis test with Dunn’s multiple comparison test, and Chi-square test or Freeman-Halton extension of the Fisher’s exact probability test as appropriate for normally or not normally distributed, and categorical data. Further *post hoc* analyses were performed using the unpaired *t*-test with Welch’s correction, and Mann–Whitney *U* test with Bonferroni correction for multiple comparisons to calculate the adjusted *p*-values (*P*_adj_), as needed.

The Friedman test was used to compare hair cortisol concentrations for the five time points within each group. Because most women did not have results for all five time points, *post hoc* analysis for the Friedman test was performed using the Wilcoxon matched-pairs signed rank test. A natural log transformation was required for the hair cortisol data prior to performing the Wilcoxon tests to best satisfy statistical assumptions. The Holm-Bonferroni correction was applied to *p*-values to correct for multiple comparisons. Additionally, the linear regression for median hair cortisol concentrations from PC to T3 for each group were compared using analysis of covariance with the Bonferroni correction for multiple comparisons to calculate the adjusted *p*-values. Univariate and multiple linear regressions were performed with the variables “consistent ICS use” (defined as use of ICS for ≥ 5 doses per week), “intranasal corticosteroid use” (yes/no), and “asthma” (yes/no) for T3 to determine if there was any influence of these variables on hair cortisol concentrations in that trimester.

Spearman correlations were calculated for the hair cortisol results with previously published confounders, including the age of hair sample, body mass index (BMI) (pre-pregnancy and at time of hair collection), number of hair washes per week, days since last washing, and PSS score. Because hair chemical treatment is a binary outcome, a point biserial correlation was performed using natural log transformed hair cortisol concentrations with concentrations ≥ 276 pmol/g removed to best satisfy the assumption of normally distributed data, which was unsuccessful for the PC concentrations in the treated asthma group. Correlations with the hair segment length were added *post hoc* when the segment lengths were finalized and some deviated from 3 cm.

In all cases, two-tailed *p*-values were calculated and considered significant if ≤ 0.05.

## Results

### Participant results and demographic comparison

Hair samples were analyzed for 118 pregnant women, consisting of 31 Controls, 31 No ICS, and 56 ICS Treated. Fourteen additional hair samples from the ICS Treated group could not be analyzed due to insufficient quantity, inaccurate segmentation, ICS being used to treat a condition other than asthma, or hair being collected too early in T1 (*i.e.*, if the hair segment was less than 2 cm) or >6 months postpartum. Some of the first hair samples analyzed had cortisol concentrations below the quantitation limit (0.33 pmol/mL) but could not be repeated due to insufficient quantity of the original hair sample; therefore, these results were recalculated using the quantitation limit. This recalculation predominantly affected the No ICS group (*n* = 5 patients/12 segments) compared to the Controls (*n* = 3 patients/3 segments) or ICS Treated (*n* = 2 patients/3 segments).

Comparisons of demographics, hair variables, and medication use among the groups are listed in Table 1. Overall, there were no significant differences among the three groups except for their use of intra-nasal corticosteroids and beta-agonists, which, as anticipated, were more frequent among women with asthma (Table 1). Associations between the hair cortisol results and factors previously reported to affect hair cortisol concentrations were further explored but no significant confounding effects were found (see Additional file 1: Table S1). Therefore, the test statistics were not adjusted for any of these factors. Of the women recruited postpartum or in T3, only one woman in the No ICS group received corticosteroids due to threatened preterm labour during the time point captured by her hair sample. It is unknown whether any of the women received hydrocortisone stress dose treatment during labour.

**Table 1:** Comparison of demographics, hair variables, and medication use among the three groups of pregnant women

	Controls (*n* = 31)	No ICS (*n* = 31)	ICS treated (*n* = 56)	*P*
*Demographics*				
Age, years, mean (SD)	33.8 (4.3)	31.6 (6.0)	33.3 (5.5)	0.240^k^
BMI, kg/m^2^, median (IQR, *n*)				
Pregnant	28.8 (25.4–32.5, 31)	28.2 (25.0–34.2, 25)	27.8 (24.7–32.2, 41)	0.716^l^
Pre-Pregnancy	24.6 (20.9–27.2, 31)	25.8 (22.8–31.3, 25)	24.8 (21.8–29.4, 42)	0.554^l^
PSS Score, mean (SD, *n*)	12 (5–26)	15 (7–9)	15 (6–23)	0.210^k^
*Birth Data* ^a^				
Gestational age, weeks, median (IQR, *n*)	39.6 (39.0–40.9, 30)	39.1 (37.6–40.6, 28)	39.1 (38.1–40.3, 51)	0.155^l^
Birth weight, kg, median (IQR, *n*)	3.41 (3.11–3.65, 31)	3.37 (2.76–3.64, 30)	3.31 (2.89–3.63, 52)	0.701^l^
*Hair Sample Variables*				
Sample age, days^b^, mean (SD)	334 (82)	325 (93)	315 (103)	0.661^k^
# Washes per week, median (IQR, *n*)	3.5 (2.5–4.5, 31)	3.0 (2.5–5.5, 24)	4.0 (3–6.6, 42)	0.141^l^
# Days since last washed, median (IQR, *n*)	1 (0–2, 29)	1 (0–1, 23)	1 (0–1, 37)	0.375^l^
Chemical treatment^c^, *n* (%)	19 (61.3)	17 (of 26, 65.4)	25 (of 47, 53.2)	0.563^m^
*Medication Use During Pregnancy*				
Inhaled corticosteroid use^d^, Yes/No	No	No	Yes	–
Oral corticosteroid use^e^, *n* (%)	0 (0)	3 (9.7)	4 (7.1)	0.280^m^
Intranasal corticosteroid use^f^, *n* (%)	3 (9.7)^h^	2 (6.5)^h^	16 (28.6)	**0.014** ^m^
Topical corticosteroid use^f^, *n* (%)	5 (16.1)	2 (6.4)	7 (12.5)	0.343^m^
Other steroid hormone use (e.g., progesterone)^f^, *n* (%)	3 (9.7)	4 (12.9)	6 (10.7)	0.867^m^
Beta-agonist^f,g^, *n* (%)	2 (6.4)^i^	24 (77.4)^j^	54 (96.4)	**<0.0001** ^m^
Number of other classes of medications used^f^, median (IQR)	2 (1–3)	2 (0–4)	2 (1–3)	0.650^l^

*BMI* body mass index, *PSS* perceived stress scale, *IQR* interquartile range, *SD* standard deviation

^a^Inclusive of data for twin births, the frequency of which was not significantly different among the groups. ^b^The age is calculated to the oldest part of the hair sample at the beginning of the preconception segment. ^c^For No ICS and ICS Treated, information regarding chemical treatment (color or relaxer) was not available for all women. The total number of women is indicated in the parentheses. ^d^Inhaled corticosteroid use includes any use within the time captured by the tested hair segment, regardless of frequency or duration. ^e^Oral corticosteroid use is reported if within one month prior to the tested hair segment. ^f^Use within the last 12 months, which may not be during pregnancy. ^g^Includes beta-agonist drugs that are short and long-acting, including use of combination inhaler products. ^h^Significantly different from ICS Treated, *P* < 0.05. ^i^Significantly different from both No ICS and ICS Treated, *P* < 0.0001. ^j^Significantly different from ICS Treated *P* < 0.01. ^k^One-way analysis of variance with Tukey’s multiple comparison test. ^l^Kruskal-Wallis test with Dunn’s multiple comparison test. ^m^Chi-squared or Freeman-Halton extension of the Fisher’s exact probability test, as appropriate. Significant *P*-values are in bold

### Hair cortisol concentrations increase during pregnancy

**Fig. 1:**
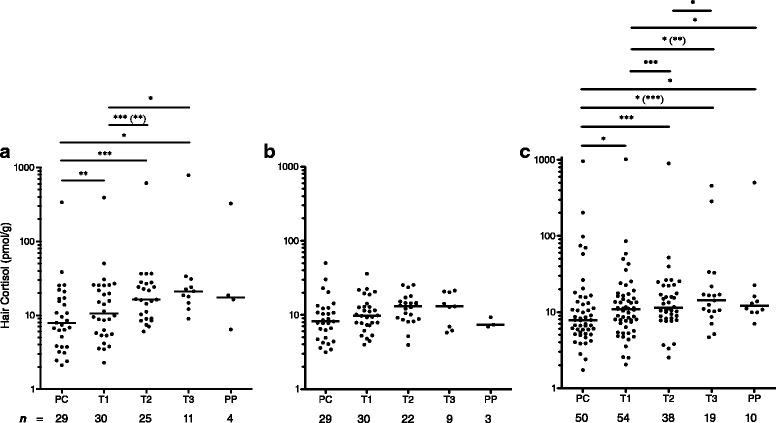
Scatter plots of median hair cortisol concentrations. Median hair cortisol concentrations (horizontal bar) in pmol/g of hair are shown for each group of pregnant women (**a** - Controls, **b** – No ICS, **c** – ICS Treated) by pregnancy time point consisting of preconception (PC), first trimester (T1), second trimester (T2), third trimester (T3), and postpartum (PP). Hair cortisol is plotted on a log10 y-axis. Sample sizes for each time point are shown below the x-axis. The change in cortisol over the five time points was significant for the Controls and ICS Treated. *Post hoc* analysis showed significant differences between time points as indicated in the figure. When analyses were repeated with all hair cortisol concentrations ≥ 276 pmol/g removed, the results were not greatly changed. The alternate *p*-values are shown in parentheses, ( ). Holm-Bonferroni correction for multiple comparisons was applied to all *p*-values. *P ≤ 0.05, **P ≤ 0.01, ***P ≤ 0.001

Hair cortisol concentrations for each time point are shown in Fig. 1. A similar increase in cortisol over the course of pregnancy from PC to T3, followed by a decline PP, was evident for all three groups, although this trend was less pronounced for the two groups of women with asthma. There were seven patients (five ICS Treated and two No ICS) with a decline in hair cortisol concentrations from PC to T2. Additionally, there was a subgroup of three women with 11 samples with hair cortisol concentrations ≥ 276 pmol/g consisting of one Control for segments PC to PP, and two ICS Treated for segments PC to T3 and T3 to PP (Fig. 1). The cortisol concentrations for the Controls differed significantly across all time points (*χ*^2^_(4)_ = 9.6, *P* = 0.028, *n* = 4) and increased from 7.9 pmol/g (IQR 3.8–17.0 pmol/g, *n* = 29) in PC to 21.1 pmol/g (IQR 14.7–31.0 pmol/g, *n* = 11) in T3. The ICS Treated group also showed overall significant changes in cortisol (*χ*^2^_(4)_ = 21.4, *P* < 0.001, *n* = 7) as well as an increase between PC (7.9 pmol/g, IQR 5.4–14.2 pmol/g, *n* = 50) and T3 (14.2 pmol/g, IQR 10.2–21.7 pmol/g, *n* = 19). Only the No ICS group did not have a significant overall change in cortisol across all time points (*χ*^2^_(4)_ = 2.1, *P* = 0.768, *n* = 3) but did show a similar trend of increasing cortisol from PC (8.2 pmol/g, IQR 4.8–12.4 pmol/g, *n* = 29) to T3 (13.0 pmol/g, IQR 8.8–15.7 pmol/g, *n* = 9), although the increase from PC to T3 was also not significant (*χ*^2^_(4)_ = 2.0, *P* = 0.583, *n* = 8). Generally, *post hoc* analyses revealed significant differences over the course of pregnancy between hair cortisol concentrations during PC and T1 compared to T2 and onward for the Controls and ICS Treated (Fig. 1).

### Hair cortisol increase during pregnancy is dampened in women with asthma

**Fig. 2:**
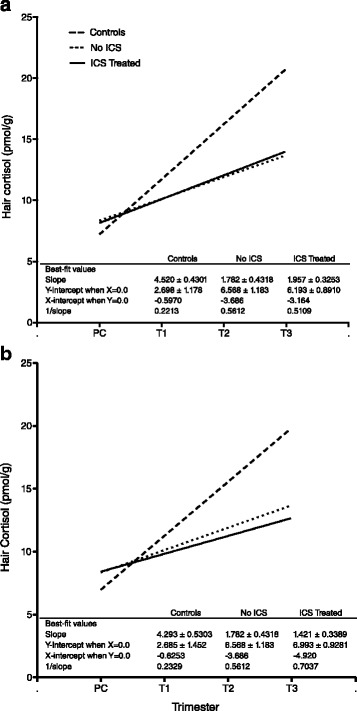
Comparison of the change in hair cortisol concentrations during pregnancy. The linear regressions for median hair cortisol concentrations for each group of pregnant women from PC to T3 are shown. The similarity of the slopes for the No ICS and ICS Treated groups can be seen. The difference of those slopes from the slope for the Controls is significant if the samples with concentrations ≥ 276 pmol/g are included (**a**), but when they are excluded (**b**), the comparison between the Controls and No ICS is no longer significant. PC = preconception, T1 = first trimester, T2 = second trimester, T3 = third trimester

When the median hair concentrations from PC to T3 were plotted for each group to determine the slopes of the regression lines for the change in cortisol during pregnancy, there was a significant difference among the three slopes (F_(2,6)_ = 14.8, *P* = 0.005) (Fig. 2a). Comparing the slopes to one another, the change in median hair cortisol concentrations from PC to T3 for the Controls was significantly different from the ICS Treated (F_(1,4)_ = 22.6, *P*_adj_ = 0.026) and No ICS (F_(1,4)_ = 20.2, *P*_adj_ = 0.033), whereas there was no significant difference between the No ICS and ICS Treated (F_(1,4)_ = 0.1, *P*_adj_ = 2.29). When the subgroup of women with samples with concentrations ≥ 276 pmol/g were excluded as potential confounders, the comparison between the Controls and No ICS was no longer significant (F_(1,4)_ = 13.5, *P*_adj_ = 0.064) (Fig. 2b).

### Hair cortisol concentrations are lower in third trimester for women with asthma

**Fig. 3:**
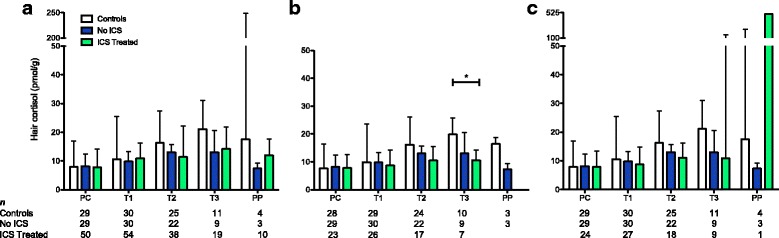
Bar graphs comparing median hair cortisol concentrations. The median hair cortisol concentrations (y-axis) at each time point (x-axis) for Controls, No ICS, and ICS Treated are shown. Sample sizes for each time point are shown below the x-axis. **a**. Comparison inclusive of all women in the ICS Treated group who reported ICS use captured in the hair sample, regardless of frequency or duration, showing no significant difference among the three groups. **b** Comparison excluding women with hair cortisol concentrations ≥ 276 pmol/g and women in the ICS Treated group who reported ICS use less than five doses per week showing a significant difference between Controls and ICS Treated women in T3. However, when the samples with concentrations ≥ 276 pmol/g were included, the comparison was no longer significant (**c**). *P ≤ 0.05, error bars indicate the interquartile range, PC = preconception, T1 = first trimester, T2 = second trimester, T3 = third trimester, PP = postpartum

All groups had a similar median hair cortisol concentration at PC and T1 with differences becoming apparent in T2 and T3 (Fig. 3a, b). When cortisol levels for each individual time point were compared among the groups, although there was a visible difference between the Controls and two asthma groups for T3, the results were not significant (*χ*^2^_(2)_ = 5.1, *P* = 0.078) (Fig. 3a) and performing the statistical analyses with and without the concentrations ≥ 276 pmol/g yielded similar results. However, if the cortisol results for all women with asthma were combined, the mean T3 cortisol concentration was significantly lower compared to the Controls (t_(34)_ = 2.189, *P* = 0.036). Furthermore, when women who reported ICS use less than five doses per week and concentrations ≥ 276 pmol/g were excluded as potential confounders, the T3 median hair cortisol concentration for the ICS Treated group was significantly lower (47 %) than the Controls (19.9 pmol/g vs. 10.6 pmol/g, U = 9, *P*_adj_ = 0.029) (Fig. 3b). Univariate and multiple linear regression analysis to determine the influence of consistent ICS use, intranasal corticosteroids, or asthma on the T3 hair cortisol concentration showed that asthma was the only significant factor (F_(1, 25)_ = 9.7, *P* = 0.005, R^2^_adj_ = 0.257). When the samples with concentrations ≥ 276 pmol/g were included (with ICS use still restricted to five or more doses per week), the median hair cortisol concentrations at T3 for ICS Treated group was still 48 % lower than the Controls, but the difference was no longer significant (21.1 pmol/g vs. 11.0 pmol/g, U = 29, *P*_adj_ = 0.386) (Fig. 3c).

## Discussion

This study is the first to investigate the use of hair cortisol as a biomarker to assess asthma and the potential effects of ICS on the HPA axis in pregnant women. The 2- to 3-fold increase in serum or salivary cortisol over the course of normal pregnancy compared to a non-pregnant state is well-documented [20–22]. Our findings, which showed an increase in hair cortisol concentrations over the course of pregnancy for all groups, are in line with previous hair cortisol research [23–25], and, specifically, the 2- to 3-fold increase in hair cortisol concentrations for the Controls corresponds well with the previous serum and saliva research. Moreover, we also found a suppressed adrenal response over the course of pregnancy in women with asthma compared to women without asthma, most notably in T3.

Our results are in contrast to findings reported previously that did not find a significant difference in serum cortisol concentrations among pregnant women with asthma, with and without ICS treatment, and pregnant women without asthma [14]. Limitations of the previous study was that serum cortisol was only assessed on one occasion per trimester for each participant in a 3-h time window, and the sample collection timing varied over a 4 to 9-week period of each trimester. Moreover, the sample collection for T3 occurred during 25–34 weeks of gestation, which is considered late T2 or early T3. This sampling pattern may not have fully captured the dynamic changes in cortisol that occur during pregnancy, including the rise in cortisol during T3 as seen in our study. Thus, our results suggest that using hair as the sample matrix, which is representative of the average cortisol levels for the entire trimester, may be more sensitive for detecting changes in cortisol between each trimester and is a significant advantage of our study.

Based on previous reports of the association between decreased cortisol production and ICS therapy [3, 12], it was anticipated that pregnant women using ICS might have decreased cortisol concentrations compared to both comparison groups. A significant decrease was apparent for women who used more than five ICS doses per week throughout pregnancy, but only in T3 when compared to Controls and if the three women with hair cortisol concentrations ≥ 276 pmol/g were excluded. Unexpectedly, the No ICS group showed a diminished adrenal response over the course of pregnancy from PC to T3 similar to the ICS Treated group. One possible explanation for the lower hair cortisol concentrations in women with asthma may be sustained overwork and resultant fatigue of the HPA axis [26]. Research shows that the initial response to stress is increased cortisol production with decreased expression of pro-inflammatory cytokines; however, with chronic exposure to stress hormones there is a decrease in immune system sensitivity and response to cortisol, ultimately resulting in increased pro-inflammatory cytokines [26]. Pregnancy generates an inflammatory state and approximately one-third of women experience increased asthma symptoms when pregnant [27]. It is possible that the added physiological stress due to pregnancy in combination with asthma, or the woman’s asthma severity or chronic state, exacerbates HPA axis fatigue through chronic exposure to stress hormones and ultimately leads to a decreased cortisol response regardless of ICS use. This is supported by the linear regression analysis of our data for T3 that indicates, between consistent ICS use, intranasal corticosteroid use, and asthma, asthma accounted for approximately 26 % of the decrease observed. Also, in support of our findings, patients with chronic asthma were previously found to have a decreased response to adrenocorticotropic hormone stimulation, but research is limited in this area [28]. Alternatively, the less-pronounced increase in hair cortisol during pregnancy in women with asthma may be due to decreased cortisol sensitivity from a reduction in glucocorticoid receptors [26], as found in children with asthma who were shown to have a 5.5-fold decrease in expression of the glucocorticoid receptor [29]. Further research comparing hair cortisol concentrations in healthy adults to those with asthma, with and without ICS treatment, may confirm whether the observed difference in cortisol concentrations is due to HPA axis fatigue from the physiological stress of pregnancy rather than asthma chronicity or severity.

The prevalence of adrenal insufficiency or suppression in pregnancy is currently not well documented. This is in part due to the dynamic and significant increases of cortisol during pregnancy potentially masking any deficit [21], and a lack of reference intervals specific to pregnancy. Previous research has largely focused on the fetal and perinatal outcomes of fetal exposure to increased maternal cortisol concentrations [30, 31], leaving a large gap in knowledge on the potential adverse effects of decreased cortisol levels. Our study suggests that women with asthma during pregnancy experience adrenal suppression, which may represent adrenal hypo-functionality that is less severe than that of adrenal insufficiency but could potentially still impact pregnancy outcomes and normal fetal maturation. Research shows that women with asthma, with or without ICS treatment, have similar perinatal outcomes to women with autoimmune adrenal insufficiency. Both groups are more likely to require a Cesarean section or have a preterm birth [32–34], potentially indicating a link with decreased cortisol levels. Our data do not suggest women with asthma experience preterm birth (Table 1), but our sample size may be too small to detect a significant difference. Yet, the results of a recent publication investigating the determinants of maternal hair cortisol at delivery support an association between preterm birth and decreased cortisol. Although they did not take asthma or other diseases into consideration, Braig et al. [35] found that women who had premature babies (≤ 37 weeks of gestation) had significantly lower hair cortisol levels in the three months prior to delivery (β = −0.16, *P* = 0.029). However, this significance did not remain when the regression model was adjusted for all other significant variables considered by the researchers (mutually adjusted β = −0.10, *P* = 0.157). Nevertheless, our research and that of Braig et al. supports the premise that hair cortisol is a useful tool for future research to ascertain whether there is an association between decreased maternal cortisol and pregnancy outcomes. We were unable to find any further research reporting on pregnancy outcomes for women with lower-than-normal cortisol production suggesting that the condition has possibly gone undetected until now, thus indicating an advantage of hair cortisol as a biomarker of adrenal function during pregnancy.

The surges in cortisol from fetal and maternal sources during pregnancy both likely contribute to fetal maturation. Multiple mechanisms, such as the release of placental corticotropin releasing hormone and adrenocorticotropic hormone, result in increased maternal cortisol as pregnancy progresses, and the surge in cortisol in late pregnancy is involved in epigenetic processes that program fetal cardiovascular, neurologic, endocrine, and metabolic systems [21, 31]. If the required surge in cortisol is diminished, as was evident in our study in women with asthma, organ systems that rely on cortisol for maturation and programming may be adversely affected. Only one study has examined the long-term effects of asthma and asthma treated with ICS on childhood disease. The Danish National Birth Cohort followed children to 6 years of age and found that children exposed to ICS *in utero* were more likely to experience ‘endocrine, metabolic disorders’ (hazard ratio (HR) = 1.84, CI_95%_ 1.13–2.99) and digestive system diseases (HR = 1.54, CI_95%_ 1.18–2.02) [36]. Secondary analyses assessing the effects of maternal asthma, combining those with and without ICS treatment, revealed an increased risk of diseases of the respiratory system (HR_adj_ = 1.43, CI_95%_ 1.34–1.52), nervous system (HR_adj_ = 1.43, CI_95%_ 1.18–1.73), and digestive system (HR_adj_ = 1.17, CI_95%_ 1.04–1.32) [37], all of which rely on cortisol for proper fetal maturation or programming [15, 30, 31]. Although our present study was not designed to draw conclusions on the effect of decreased cortisol levels and disease risk in children born to women with asthma, the growing evidence may warrant further research in this area.

Limitations of our study include its relatively small sample size and possible discrepancies in how the hair was collected (e.g., scalp location, distance from scalp). Given the observed expected change in hair cortisol concentrations over the course of pregnancy, any inaccuracy due to improperly collected samples is not obvious, nor expected to be significant. Additionally, all medication use was self-reported, either to the study personnel or a healthcare provider. Generally, women tend to reduce their ICS use during pregnancy [38, 39], and some women in our study reported this, but issues with recall may affect the reporting of ICS and other medications. Finally, a positive bias could have occurred for the recalculated cortisol concentrations that were below the method quantitation limit. This predominantly affected the No ICS group for PC, T1, and T2 and may have reduced potential differences between this group and the Controls. Although the degree of bias cannot definitively be determined, future studies involving a larger number of women would be beneficial and resolve any uncertainty.

## Conclusions

Our findings suggest that hair cortisol may be a useful biomarker of HPA axis function during pregnancy and sensitive enough to detect the effects of asthma, both with and without ICS treatment, on systemic cortisol levels. Using hair cortisol analysis, we are the first to show that pregnant women with asthma are potentially unable to mount the expected cortisol response seen in later pregnancy regardless of ICS use. Perinatal outcomes that are known to be associated with maternal asthma may thus be a result of decreased maternal cortisol that adversely impacts fetal maturation and epigenetic programming. Building upon our current work, future research on the effects of maternal cortisol levels on pregnancy outcomes could benefit from using hair cortisol analysis as an assessment tool.

## Abbreviations

BMI, body mass index; cm/mo = centimetres per month; EIA, enzyme immunoassay; HPA, hypothalamic-pituitary-adrenal axis; HR, hazard ratio; ICS, inhaled corticosteroids; IQR, interquartile range; *n*, sample size; *P*_*adj*_, adjusted *p*-value; PC, preconception; PP, postpartum; PSS, Perceived Stress Scale; SD, standard deviation; T1, first trimester; T2, second trimester; T3, third trimester

## Additional file

Additional file 1: Table S1. Table of correlations between hair cortisol concentrations and potential confounding factors. (PDF 88 kb)
