# Supplementary material for: Hair cortisol as a hypothalamic-pituitary-adrenal axis biomarker in pregnant women with asthma: a retrospective observational study
Source: BMC Pregnancy Childbirth. 2016 Jul 20;16:176. doi: 10.1186/s12884-016-0962-4 (PMC4955128; doi:10.1186/s12884-016-0962-4)
Supplement: Additional file 1: Table S1. — Table of correlations between hair cortisol concentrations and potential confounding factors. (PDF 88 kb) [file 12884_2016_962_MOESM1_ESM.pdf]

Hair cortisol as a hypothalamus-pituitary-adrenal axis biomarker in pregnant women with asthma: a retrospective observational study

Laura Smy, Kaitlyn Shaw, Ursula Amstutz, Anne Smith, Howard Berger, Bruce Carleton, Gideon Koren

Table S1. Correlations for hair cortisol concentrations with potential confounders.*

	Controls					No ICS					ICS Treated				
	PC	T1	T2	T3	PP	PC	T1	T2	T3	PP	PC	T1	T2	T3	PP
Segment length	-0.248	.	-0.279	-0.257	0.738	.	-0.283	-0.155	-0.102	.	.	-0.079	-0.112	-0.446	-0.166
	0.194	.	0.176	0.446	0.262	.	0.129	0.492	0.795	.	.	0.571	0.502	0.055	0.647
	29	30	25	11	4	29	30	22	9	3	50	54	38	19	10
Hair sample age	-0.317	-0.376	-0.392	-0.082	0.8	-0.201	-0.268	-0.393	-0.833	-1	-0.244	-0.381	-0.367	-0.458	-0.455
	0.094	0.04	0.053	0.811	0.2	0.295	0.152	0.071	0.005	.	0.088	0.005	0.023	0.049	0.187
	29	30	25	11	4	29	30	22	9	3	50	54	38	19	10
Pregnant BMI	-0.325	-0.131	-0.112	-0.497	-0.8	0.069	-0.015	0.12	-0.143	1	0.303	0.214	0.15	0.174	0.2
	0.085	0.491	0.593	0.12	0.2	0.755	0.944	0.646	0.76	.	0.065	0.184	0.437	0.553	0.747
	29	30	25	11	4	23	24	17	7	2	38	40	29	14	5
Pre-pregnancy BMI	-0.19	-0.014	-0.147	-0.664	-0.8	0.196	0.149	0.24	0.286	1	0.429	0.382	0.246	0.216	0.3
	0.323	0.941	0.483	0.026	0.2	0.37	0.488	0.353	0.535	.	0.006	0.014	0.198	0.459	0.624
	29	30	25	11	4	23	24	17	7	2	39	41	29	14	5
PSS score	0.123	0.241	0.081	-0.546	-0.4	0.563	0.707	0.371	-0.3	-1	-0.103	0.042	0.153	0.021	0.1
	0.559	0.246	0.72	0.102	0.6	0.146	0.05	0.468	0.624	.	0.667	0.853	0.571	0.948	0.873
	25	25	22	10	4	8	8	6	5	2	20	22	16	12	5
No. of hair washes per week	-0.333	-0.277	-0.276	0.038	0.738	0.178	0.257	0.395	0.698	.	-0.033	-0.027	0.051	-0.194	-0.1
	0.077	0.138	0.181	0.911	0.262	0.428	0.236	0.13	0.081	.	0.842	0.867	0.791	0.526	0.873
	29	30	25	11	4	22	23	16	7	2	39	41	29	13	5
No. days since last washing	0.365	0.175	0.03	0.103	0.775	-0.019	0.139	0.201	0.37	-1	0.062	0.01	-0.085	-0.259	-0.224
	0.056	0.374	0.885	0.763	0.225	0.935	0.538	0.473	0.47	.	0.727	0.955	0.687	0.416	0.718
	28	28	25	11	4	21	22	15	6	2	34	36	25	12	5
Chemical treatment[b]	-0.168	-0.171	-0.171	-0.331	-0.327	-0.318	-0.267	-0.152	-0.404	-0.983	-0.129	0.161	0.071	-0.108	-0.554
	0.383	0.365	0.413	0.320	0.673	0.130	0.197	0.547	0.321	0.116	0.403	0.286	0.704	0.714	0.333
	29	30	25	11	4	24	25	18	8	3	44	46	31	14	5

PC = preconception, T1 = first trimester, T2 = second trimester, T3 = third trimester, PP = postpartum, BMI = body mass index, PSS = perceived stress scale.
*Results listed top to bottom are the correlation coefficient, p-value[a], and *n*.
[a]p-values are not adjusted for multiple comparisons.
[b]Color or relaxer. All hair cortisol concentrations were natural log transformed and outliers were removed to normalize the data (which was unsuccessful for the ICS Treated PC results) in order to perform the point biserial correlation.
